# The outcomes of Ilizarov treatment in aseptic nonunions of the tibia stratified by treatment strategies and surgical techniques

**DOI:** 10.1038/s41598-020-77569-y

**Published:** 2020-11-25

**Authors:** Łukasz Szelerski, Andżelika Pajchert-Kozłowska, Sławomir Żarek, Radosław Górski, Paweł Małdyk, Piotr Morasiewicz

**Affiliations:** 1 Department of Orthopedics and Musculoskeletal Traumatology, Medical University of Warsaw, Lindleya 4, 02-005 Warsaw, Poland; 2 Department and Clinic of Orthopaedic and Traumatologic Surgery, Wroclaw Medical University, Borowska 213, 50-556 Wroclaw, Poland; 3 Department of Orthopaedic and Trauma Surgery, University Hospital in Opole, Institute of Medical Sciences, University of Opole, Witosa 26, 41-405 Opole, Poland

**Keywords:** Trauma, Risk factors

## Abstract

Nonunions of the tibia, particularly those located in the distal third of the bone, are relatively common in clinical practice. There is no gold standard for the treatment of nonunions of the tibia. The purpose of our study was to assess the results of treatment with the Ilizarov method in patients with aseptic nonunions of the tibia, depending on the employed treatment strategies and surgical techniques. A total of 75 patients with Ilizarov treatment of aseptic nonunions of the tibia were evaluated in the study. The patients’s mean age at the beginning of treatment was 46 years. The mean follow-up period was 10 years and 11 months. The evaluated patients underwent either closed technique or open technique. The operators used one of two treatment strategies: neutral fixation without compression or continued compression. The following were assessed: rates of union, ASAMI bone scores, ASAMI functional scores, treatment time, complications, duration of hospital stay. Bone union was achieved in all of the 75 evaluated patients. The results of most analyses showed no significant differences in the assessed variables, except for the ASAMI functional scores, which were higher in the group of patients who underwent closed surgery (*Me* = 6.00 vs. *Me* = 4.00). We observed better ASAMI functional score outcomes in the patients who underwent closed fixation than in the open fixation group. The different surgical techniques and treatment strategies had no effect on the number of complications, rates of bone union, length of hospital stay, duration of Ilizarov treatment, or ASAMI bone scores. For managing nonunions of the tibia we recommend the technique of closed fixation without continued compression. The Ilizarov method in the treatment of nonunions of the tibia gives good outcomes.

## Introduction

**Figure 1:**
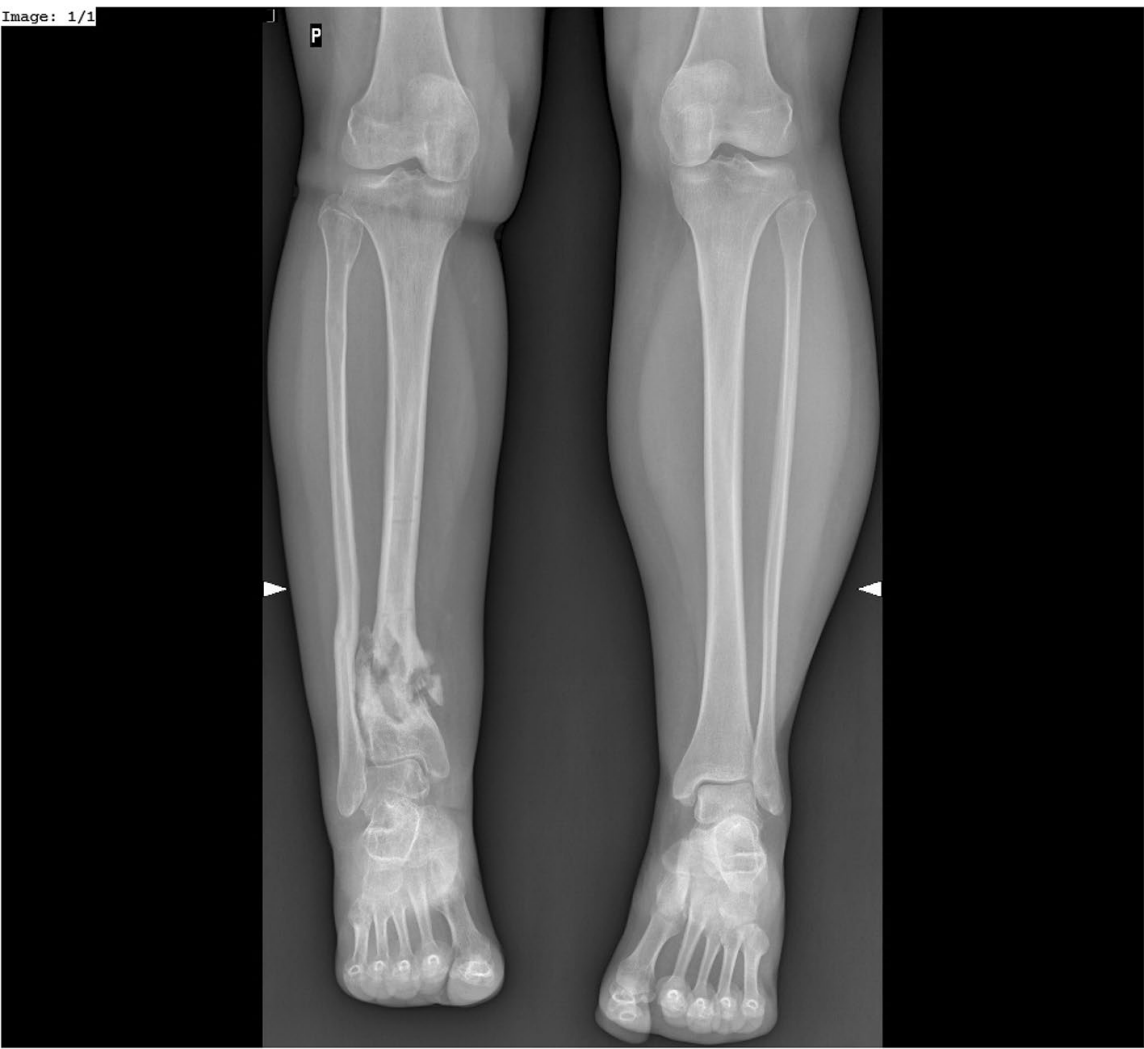
Hypertrophic non-union of 1/3 distal tibia a–p view.

Nonunions of the tibia, particularly those located in the distal third of the bone, are relatively common in clinical practice^1–4^. Despite of this, they pose a serious therapeutic challenge for orthopedic surgeons^1–3,5–14^. Nonunions of the tibia may be associated with: low-density bone tissue, bone loss, adjacent soft-tissue damage, limb shortening, limb deformities, and joint contractures (Fig. 1). All of these adversely affect the course of treatment and increase the risk of treatment failure^1–5,7–22^. In nonunions of the tibia, the Ilizarov method helps achieve bone union, eliminate possible infections, equalize limb length, and correct any deformities that may have developed over the course of treatment^1–5,7–15,17–19,21,22^.

Various strategies and surgical techniques employing the Ilizarov method have been reported for treating nonunions of the tibia^1–22^. The specific strategies or techniques are selected based on bone tissue density and vitality, limb shortening and deformity, the shape of bone fragments, condition of soft tissues, presence of infection, and operator’s preferences^1–5,7,12,14–16,20^. There is no gold standard for the treatment of nonunions of the tibia. Moreover, there are not many studies comparing the different tactics of surgical management^7,20^. Some authors claim that bone transport combined with the use of external fixators carries a higher risk of complications and yields worse outcomes in comparison with other methods of tibial nonunions treatment^2,5,11^. Most of the available analyses concern infected nonunions of the tibia^1,2,6–16,18–22^, whereas few reports discuss the treatment of tibia nonunion that is uncomplicated by infection^3–5,17^.

**Figure 2:**
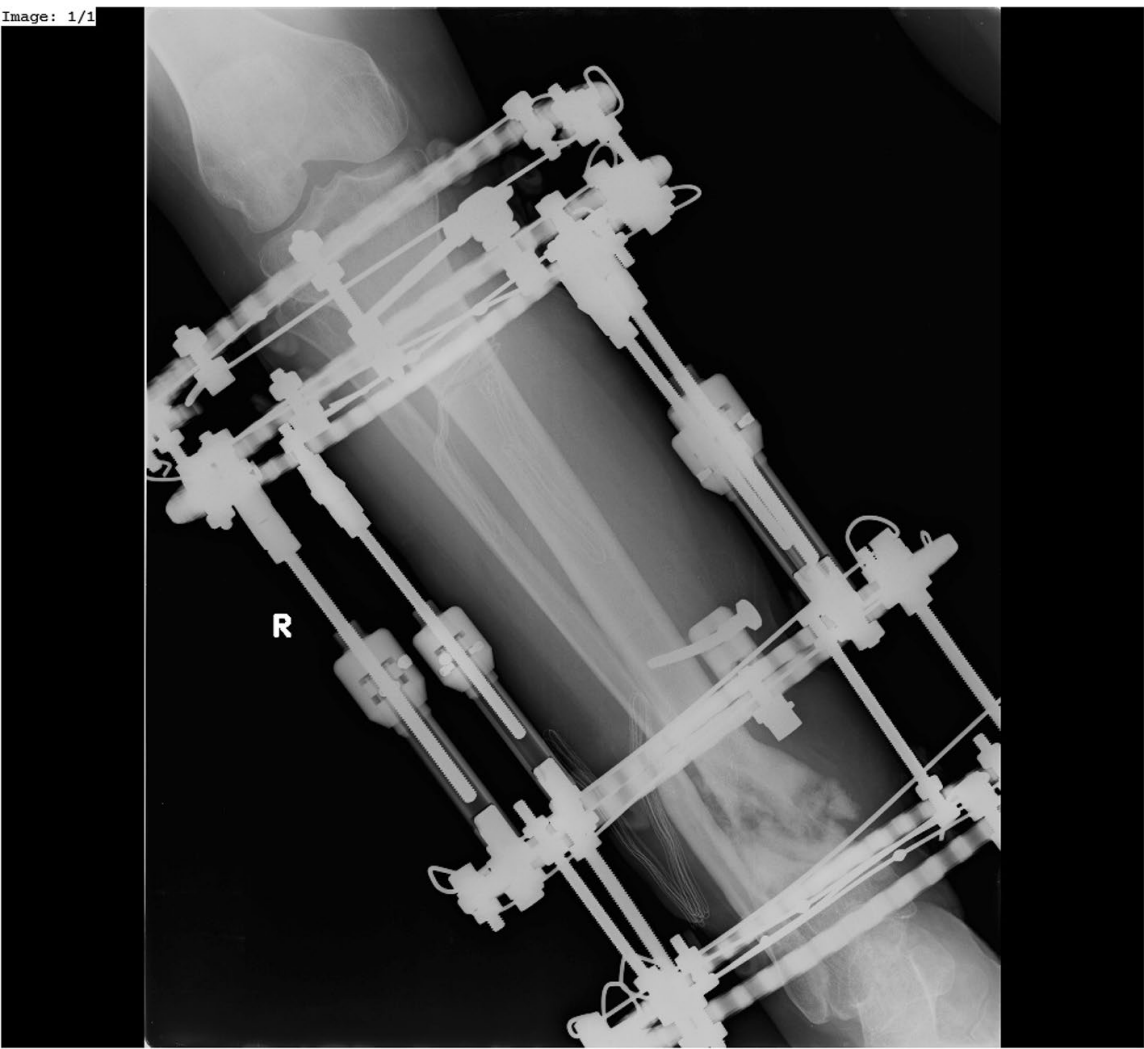
Hypertrophic non-union of 1/3 distal tibia treated by Ilizarov External Fixator (a–p view).

**Figure 3:**
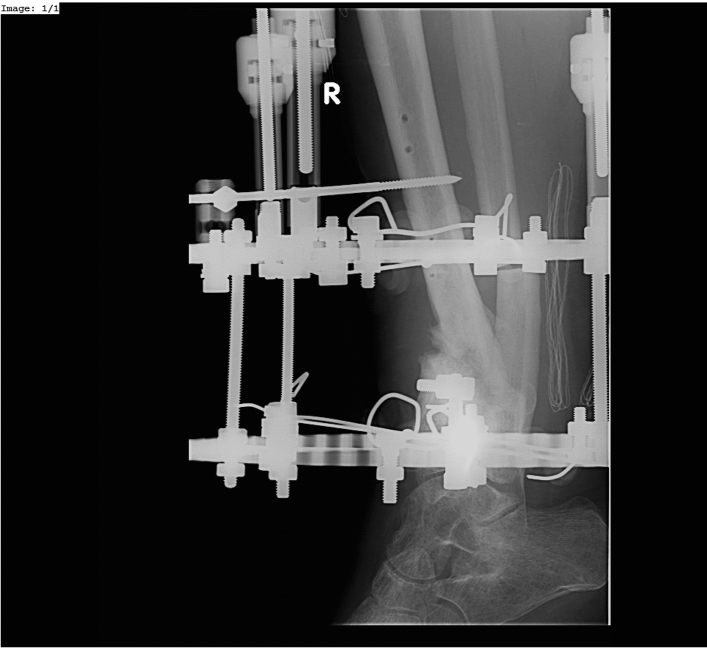
Hypertrophic non-union of 1/3 distal tibia treated by Ilizarov External Fixator (lateral view).

The purpose of our study was to assess the results of treatment with the Ilizarov method in patients with aseptic nonunions of the tibia, depending on the employed treatment strategies and surgical techniques (Figs. 2, 3).

## Materials and methods

We evaluated 125 patients with nonunions of the tibia treated with the Ilizarov method over the years 2000–2016. The inclusion criteria were the patient’s informed consent, nonunions of the tibia treated with the Ilizarov method, absence of infection, shortening of the limb < 1 cm, accessibility of complete clinical and radiographic records from the course of treatment, minimum follow-up period of 3 years after treatment completion. A total of 75 patients (23 females and 52 males) met the inclusion criteria and were evaluated in the study. The patients’ mean age at the beginning of treatment was 46 years (15–84 years). The mean follow-up period was 10 years and 11 months (ranging from 38.7 months to 19 years).

59 of them were treated operatively previously, usually by open reduction and internal fixation with three cortical screws or monolateral external fixator. A few had stabilization with intramedullary nail. In these cases we removed blocking screws and put an Ilizarov external fixator to compress the gap. 16 of them were firstly treated with the cast.

The study was approved by the Institutional Local Review Board of Warsaw Medical University. All methods were carried out in accordance with relevant guidelines and regulations. Informed consent was obtained from all subjects.

All surgical procedures were conducted by three experienced orthopedic surgeons. In the case of nonunions of the proximal and middle thirds of the tibia, the Ilizarov external fixator consisted of four rings fixed to the tibia and fibula with Kirschner wires. In the case of nonunions involving the distal tibial metaphysis or epiphysis, the Ilizarov fixator consisted of three rings (fixed to the tibia and fibula with Kirschner wires) and a foot frame stabilized with three olive wires.

Each patient admitted to the ward was carefully examined before. We checked level of C-reactive protein and procalcitonin to determine signs of active infection. We assessed the X-ray and looked for signs of sequestrum or bone necrosis. In questionable cases we ordered MRI.

The treatment of nonunions of the tibia with the Ilizarov method was conducted with various strategies and surgical techniques, selected based on the condition of bone and soft tissues, type of nonunions, shape of bone fragments, limb length discrepancy, limb deformity, and operator’s preference. The selected tactics of surgical management can be divided into two techniques and two strategies. The evaluated patients underwent either closed (technique 1) or open (technique 2, with open, small resection of bone fragments, with adaptation of the edges of the nonunion and stabilization). The operators used one of two treatment strategies: either neutral fixation without compression (strategy 1) or continued compression (adjusted by 0.25 mm every 3 days) until bone union within the location of the nonunion was achieved, as confirmed by radiographic and clinical evidence (strategy 2). All patients underwent fibular osteotomy. We used to cut fibula routinely, to ensure that during stabilization or compression of the nonunion, the fibula does not block or stiffen tibia.

The patients were also divided into subgroups based on the surgical technique. These subgroups comprised 48 patients (group 1) who underwent closed fixation of the nonunion and 27 patients (group 2) who underwent open, small resection of bone fragments, with adaptation of the edges of the nonunion and stabilization.

Divided in terms of the treatment strategy the subgroups of patients comprised 38 patients (group 1) where no compression was exerted, 20 patients (group 2) with continued compression following a closed fixation procedure, and 17 patients (group 3) with continued compression following an open fixation procedure.

The choice of technique was dependent on the type of pseudarthrosis. Hypertrophic pseudarthroses were treated with a closed technique, whereas in atrophic pseudarthroses a small incision was made to decorticate (“scarify”) the surfaces of adjacent bone fragments, followed by stabilization of the pseudarthrosis with an Ilizarov fixator. This latter technique was classified as “open”. The use of compression, and its type, (i.e. the choice of treatment strategy) depended on the operators’ individual preferences, which were influenced by our surgical team’s learning curve in conducting Ilizarov fixation.

Walking with the use of two elbow crutches was initiated on postoperative day 1. Over the course of Ilizarov treatment, patients were encouraged to bear more and more weight on the operated limb until they could discard the crutches and walk with full weight bearing. Follow-up visits, including follow-up X-rays, were scheduled in 2–6-week intervals.

The Ilizarov external fixators were removed after bone union within the nonunion was confirmed radiographically and clinically. The radiographic criterion of union was the presence of at least 3 out of 4 cortices or continuous trabecular bridging between the bone fragments in anteroposterior and lateral views. The clinical criteria were the absence of pain, absence of pathological mobility, and absence of crural deformity on fixator dynamization and forcible attempts at movement near the healing nonunion. Once the Ilizarov fixator was removed, the patients were advised to walk with the help of two elbow crutches with partial weight-bearing on the operated limb over a period of 3–6 weeks. Weight bearing was gradually increased, depending on the radiographic evidence of progress in bone remodeling at the site of pseudarthrosis.

Clinical and radiographic outcomes were assessed based on the medical records produced over the course of treatment and at the follow-up visit at least 3 year after treatment completion.

The following were assessed: rates of union, the Association of the Study and Application of the Method of Ilizarov (ASAMI) bone scores^23,24^, ASAMI functional scores^23,24^, treatment time, total number of complications per patient (refracture, secondary/persistent deformity, secondary/persistent limb length discrepancy, implant loosening and/or damage, implant-site infections, nerve damage, vascular damage, amputation, edema), duration of hospital stay.

The ASAMI bone scores were based on four parameters: infection, bone union, deformity, and limb length inequality^23,24^. The ASAMI functional scores were based on four parameters: stiff equinus foot position at the ankle joint, patient activity, significant limp, pain, and reflex sympathetic dystrophy^23,24^. The assessments were conducted in the whole study group collectively and in the individual surgical-technique and treatment-strategy subgroups separately.

Statistical analyses were conducted with STATISTICA 13.3. This software was used to obtain descriptive statistics along with Shapiro–Wilk test results for normality of distribution of all variables in the form of quantitative measurements in the groups of patients who underwent open or closed fixation procedures. Subsequently, Mann–Whitey U test and Kruskal–Wallis H test (one-way ANOVA on ranks) were used for calculating the differences between study groups. The level of statistical significance was adopted at α = 0.05; however, p-values between 0.05 and 0.1 were interpreted as showing a trend toward significance.

### Ethics approval and consent to participate

The study was approved by the Institutional Local Review Board of Warsaw Medical University. All methods were carried out in accordance with relevant guidelines and regulations.

### Consent for publication

Written informed consent was obtained from the patient for publication of this case report and any accompanying images.

## Results

Bone union was achieved in all of the 75 evaluated patients (100%). The median time to union was 203 days, with the median of 192 days for patients with hypertrophic pseudarthrosis and 301 days for patients with atrophic pseudarthrosis.

### Surgical techniques

ASAMI bone and functional scores, Ilizarov treatment duration, the length of hospital stay, and the number of complications in patients treated with the open and closed technique were presented in Tables 1 and 2.

**Table 1:** Descriptive statistics and Shapiro–Wilk test results for quantitative variables in the patients who underwent open fixation procedures (n = 27).

	*M*	*Me*	*SD*	*Sk*	Kurt	Min	Max	*S–W*	*p*-value
Length of hospital stay (days)	15.89	11.00	9.35	1.15	0.68	4.00	39.00	0.87	0.003
Ilizarov treatment duration (days)	251.11	218.00	150.29	2.16	6.62	83.00	810.00	0.82	< 0.001
ASAMI bone score	9.11	10.00	2.56	− 2.62	5.27	2.00	10.00	0.37	< 0.001
ASAMI functional score	4.89	4.00	1.01	0.24	− 2.11	4.00	6.00	0.63	< 0.001
Number of complications	0.44	0.00	0.64	1.17	0.40	0.00	2.00	0.69	< 0.001

*M* mean, *Me* median, *SD* standard deviation, *Sk.* skewness,* Kurt.*, kurtosis, *Min *minimum, *Max* maximum, *S–W* Shapiro–Wilk test result, *p-value* significance of normality of distribution.

**Table 2:** Descriptive statistics and Shapiro–Wilk test results for quantitative variables in the patients who underwent closed fixation procedures (n = 48).

	*M*	*Me*	*SD*	*Sk*	Kurt	Min	Max	*S–W*	*p*-value
Length of hospital stay (days)	14.40	10.50	11.86	2.48	8.10	4.00	68.00	0.74	< 0.001
Ilizarov treatment duration (days)	226.40	192.50	115.86	1.82	3.67	84.00	630.00	0.82	< 0.001
ASAMI bone score	9.13	10.00	2.61	− 2.73	5.85	0.00	10.00	0.36	< 0.001
ASAMI functional score	5.42	6.00	1.16	− 2.58	8.78	0.00	6.00	0.53	< 0.001
Number of complications	0.27	0.00	0.49	1.60	1.76	0.00	2.00	0.57	< 0.001

*M* mean, *Me* median, *SD* standard deviation, *Sk.* skewness, *Kurt. *kurtosis, *Min *minimum, *Max* maximum, *S–W* Shapiro–Wilk test result, *p-value* significance of normality of distribution.

A series of Mann–Whitney U tests were conducted to assess the differences in the values of selected variables (duration of hospital stay, time to union, ASAMI bone score, ASAMI functional score, number of complications) in the subgroups of patients who underwent open (n = 27) and closed (n = 48) surgery. The results of most analyses showed no significant differences in the assessed variables, except for the ASAMI functional scores, which were higher in the group of patients who underwent closed surgery (*Me* = 6.00 vs. *Me* = 4.00). Detailed data were presented in Table 3.

**Table 3:** Mann–Whitney U test results for selected quantitative variables, stratified by the surgical technique (open vs. closed) (N = 75).

	*U*	*Z*	*p*-value	*η*^2^
Length of hospital stay (days)	516.00	− 1.46	0.145	0.03
Ilizarov treatment duration (days)	576.50	− 0.78	0.433	0.01
ASAMI bone score	645.00	0.05	0.959	< 0.01
ASAMI functional score	**457.50**	**2.51**	**0.012**	0.08
Number of complications	563.00	− 1.17	0.240	0.02

*U* and *Z* Mann–Whitney U test statistics, *p-value* level of significance, *η2 (eta-squared)* a measure of effect size for Mann–Whitney U test.

### Treatment strategies

The duration of Ilizarov-method treatment, length of hospital stay, number of complications, and ASAMI bone and functional scores in patients who underwent a no-compression Ilizarov treatment, continued compression following a ‘closed’ surgery, and continued compression following an ‘open’ surgery were presented in Tables 4, 5, and 6.

**Table 4:** Descriptive statistics and Shapiro–Wilk test results for quantitative variables in the patients who underwent Ilizarov treatment with no compression, n = 38).

	*M*	*Me*	*SD*	*Sk*	Kurt	Min	Max	*S–W*	*p*-value
Length of hospital stay (days)	15.34	11.00	10.61	1.21	0.22	4.00	39.00	0.81	< 0.001
Ilizarov treatment duration (days)	255.03	204.00	155.94	1.90	3.92	84.00	810.00	0.80	< 0.001
ASAMI bone score	8.68	10.00	3.09	− 2.00	2.21	0.00	10.00	0.45	< 0.001
ASAMI functional score	5.16	6.00	1.28	− 1.93	5.42	0.00	6.00	0.63	< 0.001
Number of complications	0.32	0.00	0.53	1.40	1.13	0.00	2.00	0.61	< 0.001

*M* mean, *Me* median, *SD* standard deviation, *Sk.* skewness,* Kurt. *kurtosis, *Min *minimum, *Max* maximum, *S–W* Shapiro–Wilk test result, *p-value* significance of normality of distribution.

**Table 5:** Descriptive statistics and Shapiro–Wilk test results for quantitative variables in the patients who underwent closed fixation procedures with subsequent continued compression (n = 20).

	*M*	*Me*	*SD*	*Sk*	Kurt	Min	Max	*S–W*	*p*-value
Length of hospital stay (days)	15.05	11.00	14.42	2.83	9.77	4.00	68.00	0.67	< 0.001
Ilizarov treatment duration (days)	190.85	169.00	66.61	0.76	− 0.17	98.00	335.00	0.93	0.182
ASAMI bone score	9.20	10.00	2.46	− 2.89	7.04	2.00	10.00	0.35	< 0.001
ASAMI functional score	5.50	6.00	0.89	− 1.25	− 0.50	4.00	6.00	0.54	< 0.001
Number of complications	0.25	0.00	0.44	1.25	− 0.50	0.00	1.00	0.54	< 0.001

*M* mean, *Me* median, *SD* standard deviation, *Sk.* skewness,* Kurt. *kurtosis, *Min *minimum, *Max* maximum, *S–W* Shapiro–Wilk test result, *p-value* significance of normality of distribution.

**Table 6:** Descriptive statistics and Shapiro–Wilk test results for quantitative variables in the patients who underwent open fixation procedures with subsequent continued compression (n = 17).

	*M*	*Me*	*SD*	*Sk*	Kurt	Min	Max	*S–W*	*p*-value
Length of hospital stay (days)	13.88	11.00	6.97	1.05	0.41	4.00	30.00	0.88	0.030
Ilizarov treatment duration (days)	243.47	232.00	108.75	0.72	0.73	83.00	496.00	0.95	0.462
ASAMI bone score	10.00	10.00	0.00	0.00	0.00	10.00	10.00	0.00	–
ASAMI functional score	5.06	6.00	1.03	− 0.13	− 2.27	4.00	6.00	0.64	< 0.001
Number of complications	0.47	0.00	0.72	1.27	0.40	0.00	2.00	0.68	< 0.001

*M* mean, *Me* median, *SD* standard deviation, *Sk.* skewness, *Kurt. *kurtosis, *Min *minimum, *Max* maximum, *S–W* Shapiro–Wilk test result, *p-value* significance of normality of distribution.

A series of Kruskal–Wallis tests were conducted in order to assess differences in the values of selected variables (length of hospital stay, time to union, ASAMI bone score, ASAMI functional score, number of complications) between the groups of patients subjected to a neutral (no compression) treatment strategy (n = 38) and those subjected to compression following ‘closed’ (n = 20) and ‘open’ (n = 17) surgery. The analysis results proved not to be significant, which indicates a lack of correlation between the individual variables and the treatment technique.

## Discussion

The Ilizarov method is recommended by a number of authors for treating nonunion of the tibia, as it is highly effective in achieving bone union, treatment of a possible infection, correcting limb length discrepancy and axial misalignment, and eliminating joint contractures^1–5,7–15,17–19^.

There are various recommended treatment strategies of nonunions of the tibia employing the Ilizarov method^2–5,7–17,19–22^. Good treatment outcomes have been demonstrated with the use of various treatment strategies^2–8,11–16,18–20^. These include: fixation alone^9,12,15,17,18^; fixation and compression^7,20^; fixation, segmental resection, and bone transport^2,4,6,12,14,15,19–21^; and fixation, resection, and compression with bone transport^5,7,8,10,11,13,14,20^. Eralp observed good treatment outcomes in infected nonunions of the tibia treated by means of either combined fixation and compression or combined fixation, resection, and compression with bone transport^7^. However, various surgical techniques and treatment strategies may affect the outcomes in ways that are not known at this time. McNally et al. assessed the effect of four different treatment strategies and techniques used in infected pseudarthrosis of the tibia on treatment outcomes in 79 patients^20^. These strategies and techniques were: monofocal distraction, monofocal compression, bifocal compression/distraction, and bone transport^20^. Post-treatment infection recurrence was observed in three patients from the monofocal compression subgroup. Primary bone union rates were the lowest (73.7%) in the monofocal compression subgroup and the highest in the bifocal compression/distraction (93.8%) and monofocal distraction (96.2%) subgroups. The authors concluded by advising against the use of monofocal compression in the treatment of pseudarthrosis of the tibia^20^.

Our study demonstrated bone union in 100% of patients, which is an outcome comparable to, or even slightly better than, those reported in literature (73.7–100%) Table 7^1–13,15–22^. The treatment strategy and surgical technique showed no effect on the proportion of patients who achieved bone union in the individual subgroups.

**Table 7:** Comparison of treatment results for nonunion of the tibia.

References	Number of patients	Bone union (%)	Complications per patient
Yin et al.^1^	590	97.8	
Peng et al.^2^	58	100	0.67
Schoenleber et al.^3^	8	100	0.875
Zhang et al.^4^	25	100	0.2
Abuomira et al.^5^	55	89	1.2
Baruah et al.^6^	50	98	
Eralp et al.^7^	13	92.3	1.38
Hosny et al.^8^	11	100	1.27
Khan et al.^9^	24	87	
Madhusudhan et al.^10^	22	81.8	2.01
Magadum et al.^11^	25	96	
Meleppuram et al.^15^	42	100	1.6
Sahu et al.^16^	60	100	
Sanders et al.^17^	19	84.2	
Shahid et al.^18^	12	100	
Wang et al.^19^	15	100	
Wani et al.^12^	26	100	2.27
Yin et al.^13^	65	100	
McNally et al.^20^	79	73.7–96.2	
Dróżdż et al.^21^	54	86	
Marsh et al.^22^	46	87	
Current study	75	100	0.25–0.45

There have been no studies assessing the number of complications depending on the employed treatment strategy and surgical technique. Our study population, depending on the subgroup, developed anywhere from 0.25 to 0.47 complications per patient, which is a slightly better result than those reported in literature, which range from 0.67 to 2.27, Table 7^2–4,19^. The employed treatment strategies and surgical techniques were observed to have no effect on the mean number of complications per patient.

There are no available reports from studies assessing the duration of treatment with an external fixator stratified by different treatment strategies and surgical techniques. Overall, the mean duration of treatment ranges from 5.8 months to 13.5 months^2–6,8,9,16,19^. These figures are similar to ours. We observed no effect of the evaluated treatment strategies or surgical techniques on Ilizarov treatment duration.

ASAMI bone scores reported by Abuomira were 51% excellent, 33% good, 9% fair, and 7% poor^5^. Khan observed 25% excellent, 58.3% good, 4.2% fair, and 12.5% poor ASAMI bone scores^9^. Meleppuram achieved 60% excellent, 15% good, and 25% fair ASAMI bone scores^15^. None of the authors cited here assessed the ASAMI bone scores stratified by the employed treatment strategy and surgical technique.

The treatment strategies and surgical techniques employed in our evaluated patient population yielded no significant differences in the resulting ASAMI bone scores.

The ASAMI functional scores reported by Abuomira were 45% excellent, 38% good, 9% fair, and 7% poor^5^. Khan observed 33.3% excellent, 50% good, 8.35% fair, and 8.35% poor ASAMI functional scores^9^. Meleppuram reported 55% excellent, 30% good, 5% fair, and 10% poor ASAMI functional scores^15^. The relevant literature contains no studies assessing ASAMI functional scores stratified by the employed surgical technique and treatment strategy.

In our study, treatment strategy was observed to have no effect on the ASAMI functional score. However, when it comes to surgical techniques, the patients who underwent closed fixation achieved significantly higher ASAMI functional scores than the open-fixation patients. This may be a result of better soft-tissue and surgical-wound healing.

We are aware of the fact that treatment of infected and aseptic pseudarthroses may produce different outcomes. Unfortunately, there is a scarcity of papers addressing aseptic pseudarthrosis treatment in the available relevant literature. Our study is one of the first ones to analyze the available techniques and strategies employed in treating pseudarthroses with an Ilizarov fixator.

The available literature reports inform us that the mean length of hospital stay for treating patients with nonunions of the tibia with an external fixator ranges from 5 to 105 days^4,12,16^. These statistics are slightly worse than those achieved in our study. We observed no effect of the employed surgical technique or treatment strategy on the length of hospital stay.

The most common complication observed in our study population during treatment with an Ilizarov fixator was Kirschner wire pin tract infection. Such infections typically respond well to topical antiseptics and oral antibiotic therapy in an outpatient setting. Deep infections involving soft tissues and bone require hospitalization, surgical debridement, and Kirschner wire replacement, which significantly lengthens the healing process (median: 189.0 days vs. 248.5 days).

Bone transport is a more complex procedure than that of employing compression/distraction. There may be problems with achieving good contact of bone ends and ensuring bone union at the docking site; moreover, more complications may develop, and the duration of treatment may be longer^5^. Open fixation procedures in patients with nonunions are more complex than closed fixation. Continued compression is more bothersome for nonunions patients than neutral fixation without compression.

We observed better ASAMI functional score outcomes in the patients who underwent closed fixation than in the open fixation group.

The different surgical techniques had no effect on the number of complications, rates of bone union, length of hospital stay, duration of Ilizarov treatment, or ASAMI bone scores.

The different treatment strategies had no effect on the number of complications, rates of bone union, rates of bone union, length of hospital stay, duration of Ilizarov treatment, ASAMI bone scores, or ASAMI functional scores.

Multicenter, randomized studies are needed in order to compose the guidelines for the treatment of aseptic pseudarthroses of the tibia. Nonetheless, our study can be considered an attempt to assess various techniques and strategies in the treatment of tibial nonunion and present our team’s experiences.

For managing nonunions of the tibia we recommend the technique of closed fixation without continued compression.

Nonetheless, the use of the Ilizarov method in the treatment of nonunions of the tibia yields good outcomes irrespective of the employed surgical technique or treatment strategy.

## Data Availability

Data used in this study are available from the corresponding author on reasonable request.

## References

[1] Yin P, Ji Q, Li T, Li J, Li Z, Liu J, Wang G, Wang S, Zhang L, Mao Z, Tang P (2015). A systematic review and meta-analysis of ilizarov methods in the treatment of infected nonunion of Tibia and Femur. PLoS ONE.

[2] Peng J, Min L, Xiang Z, Huang F, Tu C, Zhang H (2015). Ilizarov bone transport combined with antibiotic cement spacer for infected tibial nonunion. Int. J. Clin. Exp. Med..

[3] Schoenleber SJ, Hutson JJ (2015). Treatment of hypertrophic distal tibia nonunion and early malunion with callus distraction. Foot Ankle Int..

[4] Zhang H, Xue F, Xiao H (2018). Ilizarov method in combination with autologous mesenchymal stem cells from iliac crest shows improved outcome in tibial non-union. Saudi J. Biol. Sci..

[5] Abuomira IE, Sala F, Elbatrawy Y, Lovisetti G, Alati S, Capitani D (2016). Distraction osteogenesis for tibial nonunion with bone loss using combined Ilizarov and Taylor spatial frames versus a conventional circular frame. Strateg. Trauma Limb. Reconstr..

[6] Baruah RK (2007). Ilizarov methodology for infected non union of the Tibia: Classic circular transfixion wire assembly vs. hybrid assembly. Indian J Orthop..

[7] Eralp İL, Kocaoğlu M, Dikmen G, Azam ME, Balcı Hİ, Bilen FE (2016). Treatment of infected nonunion of the juxta-articular region of the distal tibia. Acta Orthop. Traumatol. Turc..

[8] Hosny G, Shawky MS (1998). The treatment of infected non-union of the tibia by compression-distraction techniques using the Ilizarov external fixator. Int. Orthop..

[9] Khan MS, Rashid H, Umer M, Qadir I, Hafeez K, Iqbal A (2015). Salvage of infected non-union of the tibia with an Ilizarov ring fixator. J. Orthop. Surg. (Hong Kong)..

[10] Madhusudhan TR, Ramesh B, Manjunath K, Shah HM, Sundaresh DC, Krishnappa N (2008). Outcomes of Ilizarov ring fixation in recalcitrant infected tibial non-unions—A prospective study. J. Trauma Manag. Outcomes..

[11] Magadum MP, Yadav CM, Phaneesha MS, Ramesh LJ (2006). Acute compression and lengthening by theIlizarov technique for infected nonunion of the tibia with large bone defects. J. Orthop. Surg. (Hong Kong)..

[12] Wani NB, Syed B (2015). Ilizarov ring fixator in the management of infected non-unions of tibia. SICOT J..

[13] Yin P, Zhang L, Li T, Zhang L, Wang G, Li J, Liu J, Zhou J, Zhang Q, Tang P (2015). Infected nonunion of tibia and femur treated by bone transport. J. Orthop. Surg. Res..

[14] Cattaneo R, Catagni M, Johnson EE (1992). The treatment of infected nonunions and segmental defects of the tibia by the methods of Ilizarov. Clin. Orthop. Relat. Res..

[15] Meleppuram JJ, Ibrahim S (2016). Experience in fixation of infected non-union tibia by Ilizarov technique—A retrospective study of 42 cases. Rev. Bras. Ortop..

[16] Sahu RL, Ranjan R (2016). Treatment of complex nonunion of the shaft of the tibia using Ilizarov technique and its functional outcome. Niger Med. J..

[17] Sanders DW, Galpin RD, Hosseini M, MacLeod MD (2002). Morbidity resulting from the treatment of tibial nonunion with the Ilizarov frame. Can. J. Surg..

[18] Shahid M, Hussain A, Bridgeman P, Bose D (2013). Clinical outcomes of the Ilizarov method after an infected tibial non union. Arch. Trauma Res..

[19] Wang H, Wei X, Liu P, Fu YH, Wang PF, Cong YX, Zhang BF, Li Z, Lei JL, Zhang K, Zhuang Y (2017). Quality of life and complications at the different stages of bone transport for treatment infected nonunion of the tibia. Medicine (Baltimore)..

[20] McNally M, Ferguson J, Kugan R, Stubbs D (2017). Ilizarov treatment protocols in the management of infected nonunion of the Tibia. J. Orthop. Trauma.

[21] Dróżdż M, Rak S, Bartosz P, Białecki J, Marczyński W (2017). Results of the treatment of infected nonunions of the lower limbs using the Ilizarov method. Ortop. Traumatol. Rehabil..

[22] Marsh DR, Shah S, Elliott J, Kurdy N (1997). The Ilizarov method in nonunion, malunion and infection of fractures. J. Bone Jt. Surg. Br..

[23] Paley D, Catagni MA, Argnani F, Villa A, Benedetti GB, Cattaneo R (1989). Ilizarov treatment of tibial nonunions with bone loss. Clin. Orthop. Relat. Res..

[24] Catagni, M.A. Classification and treatment of Nonunion. In *Operative Principles of Ilizarov by ASAMI Group*. 190–198 (Williams and Wilkins, Baltimore, 1991).

